# Recruitment of high risk women for HIV prevention trials: baseline HIV prevalence and sexual behavior in the CAPRISA 004 tenofovir gel trial

**DOI:** 10.1186/1745-6215-12-67

**Published:** 2011-03-07

**Authors:** Quarraisha Abdool Karim, Ayesha BM Kharsany, Janet A Frohlich, Cheryl Baxter, Nonhlanhla Yende, Leila E Mansoor, Koleka P Mlisana, Silvia Maarschalk, Natasha Arulappan, Anneke Grobler, Sengeziwe Sibeko, Zaheen Omar, Tanuja N Gengiah, Mukelisiwe Mlotshwa, Natasha Samsunder, Salim S Abdool Karim

**Affiliations:** 1 Centre for the AIDS Programme of Research in South Africa (CAPRISA), Nelson R Mandela School of Medicine, University of KwaZulu-Natal, Durban, South Africa; 2 Mailman School of Public Health, Department of Epidemiology, Columbia University, New York, USA

## Abstract

**Background:**

Young women in sub-Saharan Africa bear a disproportionate burden of HIV infection compared to men but have limited options to reduce their HIV risk. Microbicides could fill an important HIV prevention gap for sexually active women who are unable to successfully negotiate mutual monogamy or condom use.

**Purpose:**

This paper describes the baseline sample characteristics in the CAPRISA 004 trial which assessed the safety and effectiveness of the vaginal microbicide, 1% tenofovir gel for HIV prevention in South Africa.

**Methods:**

This analysis assessed the baseline demographic, clinical and sexual behavior data of women screened and enrolled into the trial. The characteristics were summarized using descriptive summary measures; expressed as means and percent for categorical variables.

**Results:**

HIV prevalence at screening was 25.8% [95% Confidence Interval (CI):23.9-27.7). Of the 889 eligibly enrolled women who contributed follow-up data, rural participants recruited from a family planning (FP) clinic were younger, more likely to be living apart from their regular partner, reported lower coital frequency, had lower condom use (p < 0.001). In contrast, urban participants recruited from a sexually transmitted disease (STD) clinic reported higher numbers of lifetime sexual partners, new partners in the last 30 days and receiving money in exchange for sex (p < 0.001).

**Conclusion:**

The populations selected provide suitable diverse target groups for HIV prevention intervention studies.

**Trial registration:**

ClinicalTrials.gov: NCT 00441298

## Background

It is estimated that sexual transmission accounts for 85% of HIV infections and more than 50% of the global burden of infection is in women [1,2]. The majority of HIV infected women live in Sub-Saharan Africa and young women in this region have a 3-6 fold higher burden of HIV infection compared to men [1,3,4].

South Africa has the highest burden of prevalent and incident HIV infections, with the province of KwaZulu-Natal at the epicenter of the pandemic [5-10]. Pre-trial cohort studies at the Centre for the AIDS Programme of Research in South Africa (CAPRISA) Vulindlela and eThekwini clinical research sites reported high HIV incidence rates in young women aged 14-30 years utilizing public sector family planning (FP) and sexually transmitted diseases (STD) clinics [11].

The ability of women in these settings to function within the current "ABC" (Abstinence, Be faithful, Condom use) paradigm of HIV prevention is limited [12] especially for those unable to be abstinent, negotiate mutually faithful monogamy or condom use due to complex structural, social, economic and political factors and underscore the urgent need for women-initiated HIV prevention options [13,14]. Therefore, the understanding of HIV risk behaviors within target populations is an advantage as these could provide an indication of the potential differential effects of an intervention on reported behavior.

Topical microbicides offer an option for these women to reduce their HIV risk and to date several classes of products have undergone advanced clinical testing [15-20]. These trials have provided important lessons for the conduct of microbicide trials [21] and informed design, product selection, and dosing strategy in the CAPRISA Phase IIB trial which assessed the safety and effectiveness of 1% tenofovir gel in preventing HIV infection in women in KwaZulu-Natal, South Africa (CAPRISA 004 trial). The CAPRISA 004 trial recently demonstrated the groundbreaking result of an estimated 39% reduction in heterosexually transmitted HIV and tenofovir gel (p = 0.017), a promising HIV prevention biomedical intervention for women [22].

The purpose of this exploratory analysis was to assess the baseline sample characteristics of the CAPRISA 004 trial participants to optimize recruitment for HIV prevention trials.

## Methods

### Ethical approvals

The protocol for the primary study, informed consent forms and study related materials were reviewed and approved by the University of KwaZulu-Natal Biomedical Research Ethics Committee, Ref: E111/06, the Protection of Human Subject Committee in the Office of International Research Ethics at FHI Ref: 9946, and the South African Medicines Control Council (MCC), Ref: 20060835. The trial was registered with ClinicalTrials.gov, number NCT 00441298.

### Study procedures

The CAPRISA 004 trial was conducted between May 2007 and March 2010 in KwaZulu-Natal, South Africa at the CAPRISA Vulindlela Clinical research site in Vulindlela, a rural community 150 km west of Durban (rural) and at the CAPRISA eThekwini Clinical research site in the Durban city centre (urban).

At the rural site, volunteers were recruited from among women attending the FP clinic from one of the primary health care centre, adjacent to the CAPRISA Vulindlela clinical research site [11,23], whilst at the urban site volunteers were recruited from women attending the STD clinic at the Prince Cyril Zulu Communicable Disease Centre adjacent to the CAPRISA eThekwini clinical research site [11].

Participant screening, enrolment and randomization procedures have previously been described in detail [22]. Briefly, volunteers at the study sites were provided with study information in English and/or *isiZulu *and those agreeing to continue with study participation were eligible if they were 18 to 40 years of age, willing to provide written informed consent for screening, agreed to provide adequate locator information for study retention purposes, agreed to adhere to study visit schedule, were sexually active; defined as having had vaginal intercourse at least twice within the last 30 days prior to screening, HIV negative, not pregnant, agreeable to be on a non-barrier form of contraception, creatinine clearance of >50 ml/min using the Cockcroft and Gault method [24] and no evidence deep epithelial disruption on pelvic examination. Volunteers were excluded if they had plans to travel away from the study site following enrolment, planned to relocate away from the study site, planned to become pregnant, planned to or were currently enrolled in any other study of an investigational product or behavior modification related to HIV prevention, had any known allergy to latex or had an untreated sexually transmitted infection.

Through two screening visits volunteers were assessed for eligibility, contraceptive needs, pregnancy, HIV in the context of pre- and post-test counseling, renal function, physical and medical evaluations and for pre-existing deep epithelial disruption/genital ulceration by speculum-aided pelvic examination.

Within 30 days of the first screening visit, returning eligible volunteers proceeded with the enrolment and specimen storage consents, completed study enrolment procedures and were randomly assigned to receive either 1% tenofovir gel or the hydroxy-ethylcellulose (HEC) placebo in a 1:1 ratio. Participants received an assigned study gel in boxes of ten in quantities guided by the frequency of coital activity. At the enrolment visit, trained study staff collected demographic, behavioral, sexual and reproductive health data using interviewer administered standardized questionnaires. Detailed medical history was collected; physical and clinical assessments were completed. Blood was drawn for baseline safety assessments, storage and retrospectively tested for Hepatitis B and herpes simplex virus type 2 (HSV-2) antibodies.

### Follow-up care

At screening volunteers identified as HIV positive were referred to the PEPFAR-funded CAPRISA AIDS Treatment (CAT) Programme operational at both the rural and urban sites for free care, support and antiretroviral treatment. Participants with window-period HIV infection at enrolment (i.e. sero-negative but with detectable virus based on HIV-1 RNA PCR) [25] and those who became HIV infected during follow-up were referred to the CAPRISA 002 Acute HIV infection study for follow-up counseling, care and support.

### Statistical analysis and reporting

The demographic and behavioral characteristics at baseline were summarized using descriptive summary measures; expressed as means [±standard deviation (±SD)] and percent for categorical variables. Fisher's exact tests were used for the analysis of categorical data, and unpaired t-tests or the Wilcoxon rank sum tests for the analysis of continuous data. All analyses were performed using two-sided tests. The SAS statistical package (version 9.1.3; Statistical Analysis Software, North Carolina, USA) was used for analysis.

## Results

### Recruitment and eligibility

**Figure 1:**
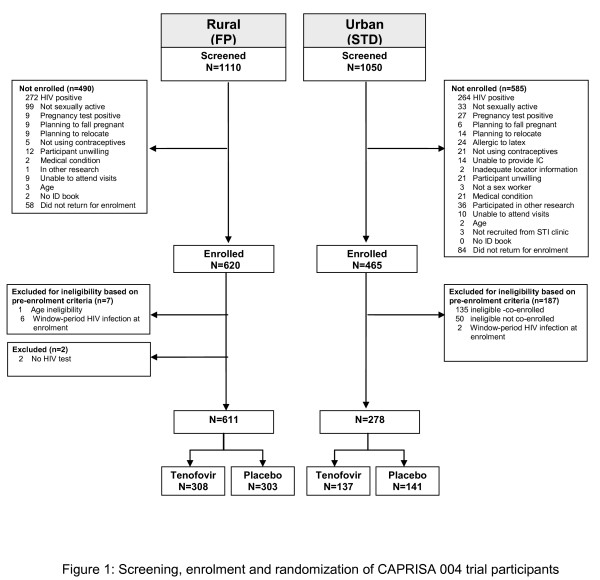
**Screening, enrolment and randomization of CAPRISA 004 trial participants**.

Participants were accrued over 19 months from May 2007 to January 2009. The total number of volunteers screened, numbers and reasons for exclusion, and total number of participants enrolled, randomized and assigned to study arm by site is presented in figure 1. Of the 2160 volunteers screened, 1110 (51.4%) were from the FP clinic at the rural site and 1050 (48.6%) from the STD clinic at the urban site. Of the 2079 participants tested for HIV, HIV prevalence at screening was 25.8% [95% Confidence Interval (CI) 23.9-27.7]; and was the most common reason for volunteer ineligibility. The age specific HIV prevalence is presented in table 1. Of the 1227 volunteer's eligible for enrolment, a total of 142 (6.6%) did not return for their enrolment visit resulting in a total of 1085 participants being enrolled and randomized.

**Table 1:** HIV prevalence at screening by age group, overall and by site

	**HIV prevalence**					
	
	**Overall**		**Rural (FP)**		**Urban (STD)**	
	**n/N**	**% (95%CI)**	**n/N**	**% (95%CI)**	**n/N**	**% (95%CI)**
	
**HIV prevalence**	536/2079*	25.8 (23.9-27.7)	272/1081	25.2 (22.6-27.9)	264/998	26.5 (23.8-29.3)
**Age specific HIV prevalence**						
**Missing**	0/2	0			0/2	0
18 to 24 years	242/1200	20.2 (18.0-22.6)	122/685	17.8 (15.1-20.9)	120/515	23.3 (19.8-27.2)
25 to 29 years	138/465	29.7 (25.6-34.1)	66/209	31.6(25.44-38.4)	72/256	28.1 [22.8-34.1)
30 to 34 years	104/261	39.9 (33.9-46.1)	59/126	46.8 (38.0-55.9)	45/135	33.3 (25.6-42.0)
35 to 40 years	52/151	34.4 (27.0-42.7)	25/61	41.0 (28.8-54.3)	27/90	30.0 (21.0-40.7)

*81 missing observations were excluded from percentage calculations.

Following enrolment, 196 (18.0%) participants were considered ineligible and excluded; 8 (6-FP rural and 2-STD urban) participants were confirmed to be within the window-period of HIV infection at enrolment; 185 participants were considered to be ineligibly enrolled according to the protocol eligibility criteria as they had participated in the last 12 months prior to enrolment (n = 50) or were currently co-enrolled (n = 135) in a trial of vaginally applied product related to HIV prevention [26], one participant provided false identification and was subsequently identified as being less than 18 years of age, two participants did not have a post-randomization HIV test that was required for endpoint ascertainment. The independent data safety and monitoring board reviewed the safety data for all ineligibly enrolled participants, found no negative safety trends and agreed to the plans for discontinuation of these participants [27]. The final number of eligible enrolled participants was 889, 611 were enrolled at the rural site and 278 at the urban site. A total of 445 participants were randomized to tenofovir gel arm and 444 to the placebo gel arm.

### Baseline Socio-demographic, Clinical and Sexual Behavioral Characteristics

The baseline socio-demographic, clinical and sexual behavioral characteristics are presented in table 2. Rural participants recruited from the FP clinic were younger, had lower mean parity and were more likely to be living apart from their regular partner. In contrast, urban participants recruited from the STD clinic were older, with higher levels of education and income. With regard to sexual behavior characteristics, rural FP participants had a lower mean age of sexual debut, reported lower mean number of sex acts in the past 7 and 30 days, were more likely to be living separately from their regular partner due to partner or self-employment options and had lower condom use compared to their urban STD counterparts. A higher proportion of urban STD participants reported having a new partner in the past 30 days, having higher numbers of lifetime sexual partners and having received money in exchange for sex compared to the rural FP participants (all p < 0.001).

**Table 2:** Baseline characteristics of enrolled participants by site^a^

**Variable**	**Variable category**	**Overall**	**Rural (FP)**	**Urban (STD)**	***P value***
					
		**N = 889**	**n = 611**	**n = 278**	
***Sociodemographic characteristics***					
Age (Mean; ± SD; range)		23.9; ± 5.1; 18-40	23.3; ± 4.9; 18-40	25.1; ± 5.4;18-39	<0.001
Parity (Mean; ± SD; range)		1.1 (0-8)	1.1 (0-5)	1.4 (0-8)	<0.001
Age groups (n, %)					
	18 to 24 years	579 (65.1)	423 (69.2)	156 (56.1)	0.001
	25 to 29 years	177 (19.9)	112 (18.3)	65 (23.4)	
	30 to 34 years	85 (9.6)	52 (8.5)	33 (11.9)	
	35 to 40 years	48 (5.4)	24 (3.9)	24 (8.6)	
Education level (n, %)					
	No Schooling	4 (0.4)	4 (0.7)	0 (0)	<0.001
	Primary school	37 (4.2)	25 (4.1)	12 (4.3)	
	High school not complete	483 (54.3)	377 (61.7)	106 (38.1)	
	High school complete	301 (33.9)	195 (31.9)	106 (38.1)	
	Some Tertiary education	64 (7.2)	10 (1.6)	54 (19.4)	
Income level (n, %)					
	No income	96 (10.9)	78 (12.8)	18 (6.5)	<0.001
	<R1000 per month	718 (80.7)	526 (86.1)	192 (69.1)	
	>R1001 per month	75 (8.4)	7 (1.1)	68 (24.5)	
Contraception (n, %)					
	Hormonal injectables	730 (82.1)	508 (83.1)	222 (79.9)	0.58
	Hormonal oral	138 (15.5)	89 (14.6)	49 (17.6)	
	Tubal ligation	20 (2.2)	13 (2.1)	7 (2.5)	
	Hysterectomy	1 (0.1)	1 (0.2)	0 (0)	
***Sexual behavioral characteristics***					
Age at sexual debut (Mean, ± SD, range)		17.4; ± 2.0; 12-26	17.3; ± 2.0; 12-25	17.7; ± 2.0; 12-26	0.01
Total lifetime sex partners (Mean, ± SD, range)		3.3; ± 10.5; 1-202	2.1; ± 1.2; 1-10	6.0; ± 18.4; 1-202	0.001
Sex acts in the past 7 days (Mean, ± SD, range)		2.0; ± 3.0; 0-40	1.7; ± 2.1; 0-28	2.7; ± 4.2;;0-40	<0.001
Sex acts in the past 30 days (Mean, ± SD, range)		8.4; ± 9.1; 0-130	6.7; ± 5.2; 0-40	12.1; ± 13.6;0-130	<0.001
Any anal sex in the past 30 days (n, %)		4 (0.5)	3 (0.5)	1 (0.4)	1.0
One stable sex partner in the past year (n, %)		846 (95.2)	588 (96.2)	258 (92.8)	0.04
One stable sex partner In the past 30 days (n, %)		855 (96.2)	590 (96.6)	265 (95.3)	0.45
Living with regular partner (n, %)		109 (12.3)	58 (9.5)	51 (18.3)	<0.001
New sex partner in the last 30 days (n, %)		10 (1.1)	3 (0.5)	7 (2.5)	0.01
More than 2 casual sex partners in the past year (n, %)		38 (4.3)	4 (0.7)	34 (12.2)	<0.001
Work requires sleeping overnight away from regular partner (n, %) ^b^		214 (24.1)	169 (27.7)	45 (16.4)	<0.001
Nights sleeping away from partner in the last 30 days (Mean, ± SD, range)		22.4; ± 8.3; 0-30	24.6; ± 5.9; 0-30	17.3; ± 10.5; 0-30	<0.001
Ever forced to have sex with anyone (n, %)		6 (0.7)	1 (0.2)	5 (1.8)	0.01
Ever received money in exchange for sex (n, %)		17 (1.9)	2 (0.3)	15 (5.4)	<0.001
Knows sex partner has other sex partners in the last 30 days (n, %)		179 (20.1)	115 (18.8)	64 (23.0)	0.21
Knows sex partner had an HIV test in the last 30 days (n, %)		19 (2.1)	16 (2.6)	3 (1.1)	0.09
Alcohol usually consumed by partner before sex (n, %)		262 (29.5)	162 (26.5)	100 (36.0)	0.01
Alcohol consumed by partner and self before last sex (n, %)		36 (4.0)	4 (0.7)	32 (11.5)	<0.001
Male condoms use (n, %)					
	Always	259 (29.1)	140 (22.9)	119 (42.8)	<0.001
	Sometimes	482 (54.2)	342 (56.0)	140 (50.4)	
	Never	148 (16.7)	129 (21.1)	19 (6.8)	
***Clinical characteristics***					
Genital symptoms^$ ^in the last 30 days (n, %)					
	Any symptoms	335 (37.8)	189 (30.9)	146 (52.5)	<0.001
HSV-2 prevalence		454 (51.4) ^d^	289 (47.6) ^c^	165 (59.6) ^b^	0.001

^a ^Parts of the data reported in Table 2 have previously been reported [22]^b^1 observation missing ^c ^4 observations missing ^d ^5 observations missing - Missing values were excluded from percentage calculation ^$ ^Genital symptoms of vaginal burning, pruritis, pain after sex, intermenstrual bleeding or burning and frequency when urinating or abnormal vaginal discharge or genital ulceration

Overall, 29.1% of participants reported consistent male condom use at baseline; 0.7% reported coerced sex and 1.9% reported transactional sex. About one in five (20.1%) participants reported being aware that their sex partner had other sex partners in the past 30 days and only 2.1% reported that their partner had an HIV test in the past 30 days. Although 29.5% of participants' reported that their partner usually consumed alcohol before sex, only 4.0% reported having consumed alcohol before their last sex act. All the women reported engaging in vaginal sex practice, with 4.7% also reporting oral sex and <1% reporting anal sex.

About 2 in 5 participants (37.8%) reported at least one genital symptom including symptoms of abnormal vaginal discharge being the commonest in 32.4%, followed by vaginal burning, pruritis, burning on and frequent urination, genital ulceration, pain during sex and intermenstrual bleeding, with 7.6% reporting more than one genital symptom. The prevalence of these genital symptoms was higher among urban STD (52.5%) compared to rural FP participants (30.9%). Amongst urban STD participants the proportion of women with genital symptoms ranged from 56.9% in the 25 to 29 year age group to 45.5% in the 35 to 40 year age group years, whilst amongst rural FP participants, the proportion of women with these symptoms ranged from 33.9% in the 25 to 29 year age group to 16.7% in the 35 to 40 year age group. The overall prevalence of HSV-2 antibodies at baseline was 51.4%; amongst rural FP participants it was 47.6% compared to 59.6% among urban STD participants (p = 0.001).

## Discussion

The very different rural FP and urban STD participants who comprise the CAPRISA 004 trial cohort has provided important safety and effectiveness information in terms of varying coital frequency, frequency of gel use and gel use in the presence or absence of sexually transmitted infections [22].

For Phase IIb/proof of concept HIV prevention trials it is important to identify populations at highest risk of acquiring HIV infection [21]. Data from multiple sources confirm that KwaZulu-Natal, South Africa is at the epicenter of the HIV pandemic [1,5,10,11,28]. In mature, generalized hyper-endemic settings where HIV prevalence is unprecedentedly high as in KwaZulu-Natal, identifying most at-risk populations is a challenge as it defies typical high HIV risk definitions applicable in more concentrated and low prevalence settings. Despite rural FP and urban STD population differences in socio-demographic, sexual behaviorial and clinical characteristics, these characteristics were similar to those of self-identified sex workers and urban and rural women in KwaZulu-Natal [11,29]. These pre-trial cohorts demonstrated HIV incidence rates in excess of 6.0 per 100 women years, making it reasonably easy to recruit women from specific sites. Importantly the CAPRISA 004 trial cohort epitomizes the generalized nature of the HIV epidemic in KwaZulu-Natal, the non-specific nature of HIV risk and the continued high HIV incidence rates in these settings with high HIV prevalence.

While there are clear ethical and scientific imperatives in undertaking HIV prevention trials in this setting, and identifying the populations who are still HIV uninfected but at high risk of getting infected, declines in incidence rates over time may lead to futile results [21]. Given the high incidence rates observed in young rural FP and urban STD women in the pretrial cohorts, the strategic selection, recruitment and inclusion of young women who are at high risk of HIV acquisition in HIV prevention trials is justified [11].

The diversity of sample population selected for the CAPRISA 004 trial results with young rural FP women with low coital frequency but social instability resulting from partner's being migrant workers on one hand and on the other hand, the slightly older urban STD cohort, more likely to have multiple partners, higher coital frequency and more sexually transmitted infections provides valuable safety assessment information in relation to timing and frequency of product use. Furthermore, assessing safety and effectiveness in cohorts at high risk of acquiring HIV but with varying risk factors including coital frequency, partner characteristics, risk of sexually transmitted infections, condom use patterns and study product use provides an optimal combination of circumstances and populations where study product will be used. The few years of education, limited employment opportunities and low income even if employed, force women to have men in their lives to ensure their survival. Awareness of partner HIV status including HIV risk behaviors and its implications for personal risk is understood but ability to negotiate mutually faithful monogamy and condom use remains limited. Whilst coerced sex was uncommon, the overall male condom use reporting rate of 29.1% is indicative of women's self report to study staff in the context of HIV risk-reduction counseling. However, measurement of male condom use is important, though social incohesion is an important factor contributing to the rapid spread of HIV in urban and rural settings in South Africa.

Whilst a key limitation of this study is the sample populations selected which may limit generalizability, a sector of women at high risk of acquiring HIV infection is represented. These urban STD and rural FP women enrolled in the CAPRISA 004 trial, equally randomized to receive study product and placebo, together with the HIV specific nature of the product being tested, namely 1% tenofovir gel is an optimal combination to maximize outcomes in a proof of concept trial. However, to increase representativeness, female sex workers, and females injecting drugs users and young women from the general population must be included in HIV prevention intervention trials.

## Conclusion

The baseline characteristics of enrolled rural FP and urban STD women differ significantly. The frequent and infrequent gel use based on coital activity would advance our knowledge on the safety and effectiveness of 1% tenofovir gel for male to female sexual transmission of HIV. The role of microbicides in this setting is critical to ensure the right of women to remain HIV uninfected which is key to altering HIV epidemic trajectory.

## Abbreviations

CAPRISA: Centre for the AIDS Programme of Research in South Africa; CAT: CAPRISA AIDS Treatment; CI: Confidence Interval; FHI: Family Health International; FP: Family Planning; HEC: Hydroxy-ethylcellulose; HIV: Human Immunodeficiency virus; HSV-2: herpes simplex virus type-2; PCR: Polymerase chain reaction; RNA: Ribonucleic acid; SD: Standard deviation; STD: Sexually transmitted disease

## Competing interests

Quarraisha Abdool Karim is the Co-Principal investigator of the HPTN Prevention Leadership Group (NIH/NIAID U01 AI068619) and Salim S Abdool Karim is the Protocol Chair of the HPTN 035 trial which was supported by the National Institutes of Health (grant # U01AI46749 and U01AI068633). All other authors declare that they have no competing interests.

## Authors' contributions

QAK and SSAK as Co-Principal Investigators conceived and designed the trial, oversight and implementation of the trial; responsible for critical edits and final approval of the manuscript. ABMK contributed to the analysis, drafting and writing the manuscript. NY contributed to the analysis and critical edits to the manuscript. AG as the trial statistician contributed to the design and statistical framework. CB and LEM contributed to study oversight and critical edits to the manuscript. JAF, KM, SM, NA, SS, ZO, MM, TNG, NS contributed to the site implementation of the trial, oversight, quality assurance and critical review of the manuscript. All authors have read and approved the final manuscript.
